# Current and Prospective Treatment of Adenomyosis

**DOI:** 10.3390/jcm10153410

**Published:** 2021-07-30

**Authors:** Fady I. Sharara, Mira H. Kheil, Anis Feki, Sara Rahman, Jordan S. Klebanoff, Jean Marc Ayoubi, Gaby N. Moawad

**Affiliations:** 1 Department of Obstetrics and Gynecology, The George Washington University Hospital, Washington, DC 20037, USA; Fsharara@aol.com (F.I.S.); sara.rahman@gmail.com (S.R.); 2 Virginia Center for Reproductive Medicine, 11150 Sunset Hills Rd., Suite 100, Reston, VA 20190, USA; 3 Faculty of Medicine, American University of Beirut, Beirut 11-0236, Lebanon; yousef.hibaoui@unifr.ch; 4 Department of Obstetrics and Gynecology, Cantonal Hospital Fribourg, 1702 Fribourg, Switzerland; Anis.Feki@h-fr.ch; 5 Department of Obstetrics and Gynecology, Main Line Health, Wynnewood, PN 19096, USA; jsk5068@gmail.com; 6 Department of Obstetrics and Gynecology and Reproductive Medicine, Hopital Foch, 92150 Suresnes, France; jm.ayoubi@hopital-foch.com; 7 Faculty of Medicine, University of Versailles Saint-Quentin-en-Yvelines, Université Paris-Saclay, 78000 Versailles, France

**Keywords:** adenomyosis, fertility, medical treatment, surgical treatment

## Abstract

(1) Background: Adenomyosis is a poorly understood entity which makes it difficult to standardize treatment. In this paper we review and compare the currently approved medical and surgical treatments of adenomyosis and present the evidence behind them. (2) Methods: A PubMed search was conducted to identify papers related to the different treatments of adenomyosis. The search was limited to the English language. Articles were divided into medical and surgical treatments. (3) Results: Several treatment options have been studied and were found to be effective in the treatment of adenomyosis. (4) Conclusions: Further randomized controlled trials are needed to compare treatment modalities and establish a uniform treatment algorithm for adenomyosis.

## 1. Introduction

Adenomyosis is defined as abnormal implantation of endometrial tissue into the myometrium associated with enlarging of the uterus [1]. The exact etiology of adenomyosis remains unclear with some theories suggesting invagination of the endometrium into the myometrium and others favoring metaplasia of stem cells [2]. Newer theories on the pathophysiology of endometriosis may be leading the way to changing our understanding of adenomyosis as well. Endometriosis has been described as “a fibrotic condition in which endometrial stroma and epithelium can be identified” [3]. The hypothesis is that genetic-epigenetic changes play a role in intracellular aromatase activity and result in intracellular estrogen production leading to and inflammatory, fibrotic endometrial-like tissue outside the uterus [4]. Clinically, adenomyosis commonly manifests with debilitating symptoms including menorrhagia, chronic pelvic pain, dysmenorrhea, and infertility, necessitating treatment [5]. Due to the poorly understood pathophysiology and nature of this disease, management has not been standardized and there are currently no guidelines that prioritize one treatment modality over the other [6]. Throughout the years, adenomyosis has been managed both medically and surgically, sometimes sacrificing the fertility of the patient [7]. Until recently, hysterectomy has been the only definitive treatment for patients with adenomyosis who have completed child-bearing [8]. More recently, other treatment options have been evaluated. Adenomyosis, like endometriosis, is an estrogen responsive condition [9]. This has been the basis for medical treatments that aim to regress the adenomyotic lesions by controlling the hormonal medium [9]. On the other hand, surgical approaches, other than total hysterectomies, include physically removing tissue where pathology is present, or disrupting the blood flow to the affected area [10]. Surgical techniques that preserve fertility have been developed to avoid hysterectomies in younger women [11]. There are currently no agreed-upon guidelines to follow when managing endometriosis. The National Institute for Health and Care Excellence recommends using a hormonal intrauterine device as first line treatment for adenomyosis [12]. However, to date, management of adenomyosis remains highly individualized depending on the age, symptoms, and future desire for fertility. In this paper, we review the current treatment options for adenomyosis, and compare their efficacies in controlling the condition.

## 2. Methods

This is a narrative review. A PubMed search was conducted using the terms “adenomyosis” and “treatment” to identify the different treatment modalities for adenomyosis. Further detailed searches were conducted for each of the treatments separately using the keywords “adenomyosis”, “medical treatment”, “surgical treatment”, “non-steroidal anti-inflammatory”, “combined oral contraceptives”, “gonadotropin-releasing hormone agonist”, “gonadotropin-releasing hormone antagonist”, “danazol”, “dienogest”, “aromatase inhibitor”, “ulipristal acetate”, “antiplatelet therapy”, “dopamine agonist”, “oxytocin antagonist”, “hysterectomy”, “uterus-sparing resection”, “uterine artery embolization”, “radiofrequency ablation” and “high-intensity focused ultrasound”. The search was limited to English and included articles from 2000 till May 2021. Abstracts were screened to select relevant studies. Inclusion criteria were randomized controlled trials, case controls, cohorts, case series, case reports as well as systematic reviews and meta-analyses. Exclusion criteria were any language other than English, letters to editors and video articles.

## 3. Reslts

The initial search yielded a total of 2807 articles. After limiting to the English language and to the year 2000 onwards, this number decreased to 2125. The papers were screened, duplicates were removed and the relevant articles to our research question were selected. As a result, a total of 80 articles pertaining to the different treatments of adenomyosis were included in this review.

### 3.1. Medical Management

#### 3.1.1. Non-Steroidal Anti-Inflammatories (NSAIDs)

In patients presenting with dysmenorrhea, NSAIDs have been proven to control the pain by decreasing the production of prostaglandins [13]. These medications provide only symptomatic treatment and have no effect on the adenomyotic process.

#### 3.1.2. Oral Contraceptive Pills and Gonadotropin-Releasing Hormone (GnRH) Agonists

Combined oral contraceptive pills have been proven to be effective in the treatment of dysmenorrhea [14]. Due to the lack of trials on patients with adenomyosis, data has been extracted from trials on other diseases with concomitant adenomyosis, such as endometriosis and leiomyoma uteri [15]. Suppressive hormonal therapies have been used to temporarily induce regression of adenomyosis and improve symptoms [16]. In a case series, Mansouri et al. showed regression of adenomyosis on magnetic resonance imaging (MRI) after treatment with a course of oral contraceptive pills in one patient and resolution of adenomyosis on imaging and of chronic pelvic pain clinically after treatment with leuprolide acetate, a GnRH agonist, in 3 other patients [17]. In a systematic review of RCTs by Brown et al., GnRH agonist therapy was found to be superior to no treatment and placebo, but there was no statistical difference between GnRH agonist and danazol for dysmenorrhea, or GnRH agonist and levonorgestrel for overall pain [18]. More adverse effects were reported in the GnRH agonist group, which might be a factor in limiting its use [18].

#### 3.1.3. GnRH Antagonists

There are two case reports on the use of GnRH antagonists for treating adenomyosis. Donnez et al. reported a case of a patient who was prescribed Linzagolix, a GnRH antagonist for adenomyosis after failing a course of ulipristal acetate [19]. Linzagolix significantly reduced adenomyotic lesion size and improved the patient’s dysmenorrhea and quality of life [19]. Similarly, Kavoussi et al. reported a case of a 41 year old patient who presented with a fundal adenomyoma that regressed in size after treatment with Elagolix, another GnRH antagonist [20]. The patient also reported improvement in her clinical symptoms and resolution of her pelvic pain while on this regimen [20]. These observations make it worthy to further look into GnRH antagonists as a prospective treatment option for adenomyosis.

#### 3.1.4. Levonorgestrel-Releasing Intra-Uterine Device (LNG-IUD)

Levonorgestrel IUDs were originally designed for long term contraception but have been used for their other non-contraceptive health benefits, such as control of dysmenorrhea and menorrhagia [21]. An open, randomized, observational study of 95 women evaluated the efficacy of levonorgestrel IUD after endometrial resection for treatment of menorrhagia caused by adenomyosis, and found a significantly higher rate of amenorrhea in the IUD group compared to control (100% vs. 9% respectively, *p* < 0.001) [22]. LNG-IUD has also been shown to be a promising alternative for hysterectomy in the treatment of adenomyosis [23]. The LNG-IUD can help in the management of adenomyosis by both decidualization and atrophy of the endometrium and by downregulating estrogen receptors due to high progestin release [24,25]. In a study by Ozdegirmenci et al., LNG-IUD use was compared to hysterectomy in adenomyosis and hemoglobin levels measured after treatment with either modality at 6 months then at 1 year were found to be comparable across both groups [23]. In an RCT, Shaaban et al. found the LNG-IUD to be superior to treatment with the combined oral contraceptive pill in reduction of pain in patients with adenomyosis (6.23 to 1.68 in the IUD group vs. 6.55 to 3.9 in the OCP group) [26]. Although collectively more significant in the IUD group, bleeding pattern improved in both treatment arms portrayed by a decrease in the mean number of bleeding days per month (from 9.81 to 2.63 in IUD group vs. from 9.97 to 5.52 in the OCP group), decreased number of sanitary pads used per day (6.29 to 2 in the IUD group vs. 6.13 to 3.58 in the OCP group), and an increased number of bleeding-free days at 6 months of treatment (19.32 to 25.39 in the IUD group vs. 19.1 to 23.65 in the OCP group) [26]. Uterine volume was also shown to decrease in both treatment arms but more significantly in the LNG-IUD group (10.23 to 7.63 mL with the use of IUD versus 10.42 to 8.32 in the OCP group) [26]. In a recent systematic review by Abbass et al., LNG-IUD was found to be a highly effective option for the management of adenomyosis [27]. The overall effect estimates showed that the use of LNG-IUD leads to significant reduction of pain scores starting at 3 months (standard mean difference [SMD] −1.91, *p* = 0.002) and persisting at 36 months (SMD −3.81, *p* < 0.001). Similar trends were noted when assessing heavy menstrual bleeding (SMD −3.58, *p* < 0.001 at 3 months, SMD −2.32, *p* < 0.001 at 36 months), and uterine volume (SMD −0.47, *p* < 0.001 at 6 months, SMD −0.42, *p* < 0.001 at 36 months) [27]. Hemoglobin levels were also shown to significantly increase after treatment with the hormonal IUD at 6 months (SMD 1.71, *p* < 0.001) continuing till 12 months after insertion (SMD 1.6, *p* = 0.004) [27].

#### 3.1.5. Danazol

Danazol is an androgenic hormone used in the treatment of endometriosis to shrink the ectopic endometrial tissue. Igarashi et al. extended the use of this hormone in adenomyosis and tested out the use of a danazol-loaded IUD in patients with adenomyosis [25]. In 3 different trials, the danazol IUD was inserted in adenomyotic uteri and was found to improve dysmenorrhea and decrease myometrial thickness, with significantly less side effects given the lower serum concentrations compared to oral danazol [28,29,30]. Shawki and Igarashi also showed a possible positive effect on improved fertility after removal of danazol IUD [30].

#### 3.1.6. Dienogest

Dienogest is a selective synthetic oral progestin that has been shown to improve primary and secondary dysmenorrhea [31]. In a pilot study by Hirata et al., patients with adenomyosis were administered oral Dienogest on days 2–5 of menstruation and followed up every 8 weeks after beginning treatment [32]. Their results revealed a statistically significant decrease in the visual analog scale scores of dysmenorrhea (79.6 to 9.6), chronic pain (51.6 to 9.2), and dyspareunia (27.8 to 12.5) [32]. However, hemoglobin levels were similar (11.6 to 12.3) and total uterine size and adenomyotic lesions size did not differ significantly before and after treatment (285.4 to 259.8 and 116.9 to 111.9, respectively) [32]. Adverse effects reported include worsening anemia due to metrorrhagia and mild hot flashes [32]. In another RCT of 67 patients by Osuga et al., patients who received oral dienogest treatment for adenomyosis reported a significant decrease in pain scores (−3.8 vs. −1.4, *p* < 0.001) and visual analog scales (−58.4 vs. −20.6, *p* < 0.001) but no significant difference in uterine size reduction (20 vs. 9.6, *p* = 0.103) [33]. The most frequent adverse reactions reported were irregular uterine bleeding and hot flashes [33]. Neriishi et al. conducted a retrospective cohort to address the tolerability of dienogest use for more than 2 years and found that long term use of dienogest may be associated with a decrease in uterine size (38.7 to 26.9 cm^2^, *p* < 0.01), suggesting that it may be a tolerable alternative treatment option for patients with adenomyosis [34].

When compared to triptorelin acetate, which is a GnRH agonist, in a clinical trial by Fawzy et al., dienogest was inferior in decreasing uterine size (*p* = 0.4822 compared to *p* = 0.0108), and in achieving amenorrhea (21.2% compared to 94.4%). However, both treatments demonstrated comparable significant reductions in pelvic pain (21.7 in dienogest group vs. 24.5 in triptorelin, *p* = 0.5076), and dyspareunia (20.7 in dienogest vs. 25.8 in triptorelin, *p* = 0.3899) [35]. In an RCT, Hassanin et al. compared dienorgest and OCP use in adenomyosis and found both treatments to be effective, but dienogest was superior in decreasing pain scores (3.21 vs. 4.92) and had a higher rate of side effects [36]. Furthermore, when combined with microwave endometrial ablation, dienogest has also shown a statistically significant improvement in the visual analog scale and hemoglobin levels [37].

#### 3.1.7. Aromatase Inhibitors

Aromatase inhibitors work by halting the production of estrogen, which explains their use in adenomyosis to suppress the hormonal medium which favors the progression of the disease. In a randomized controlled trial (RCT) by Badawi et al., GnRH agonists and aromatase inhibitors were shown to be equally effective in reducing adenomyosis and improving clinical symptoms [38]. Patients with adenomyosis were randomly allocated to receive letrozole, an aromatase inhibitor or goserelin, a GnRH agonist, and outcomes were reported as uterine and adenomyoma volumes at 4, 8 and 12 weeks. Results showed a comparable decrease in uterine volumes (20.1, 15.4 and 13 cm^3^ in letrozole group vs. 21.7, 15.1 and 11.7 cm^3^ in goserelin group, at 4, 8 and 12 weeks respectively), and adenomyoma volumes across both groups suggesting that aromatase inhibitors are as effective as GnRH agonists in reducing progression and improving symptoms of adenomyosis [38]. When combined together, aromatase inhibitors and GnRH analogues seemed to reduce uterine volume by around 60% at 8 weeks as shown on imaging in a case report by Kimura et al. [39].

#### 3.1.8. Ulipristal Acetate

Ulipristal is a potent progesterone receptor modulator. Its effect in adenomyosis however is inconsistent. In a retrospective, observational study by Gracia et al., premenopausal women with concomitant adenomyosis and uterine myomas were compared to a group of patients with myomas only after treatment with 12 weeks course of ulipristal acetate [40]. Amenorrhea (90.4% vs. 77.6%, *p* = 0.0017), optimal bleeding control (90.2% vs. 73.8%, *p* = 0.028) and self-reported visual analog scale scores (*p* = 0.017), were significantly higher in the group of patients with adenomyosis suggesting that ulipristal acetate might be a good treatment option for this condition [40]. Similar results were reported in an RCT by Capmas et al. in which 30 women with adenomyosis were treated with ulipristal and compared to a control group of 10 patients with the condition treated with placebo [41]. 95.24% of patients in the ulipristal group had a pictorial blood loss assessment chart (PBAC) score of less than 75 after treatment, compared to >100 before treatment (*p* < 0.01) [41]. A significant decrease in pain was also noticed in the ulipristal group at 13 weeks (*p* < 0.01), but no significant difference in pain or PBAC score was found at 6 months [41]. In contrast, there have been some reports of worsening of adenomyosis with the use of ulipristal. In an observational study by Conway et al., 6 women treated with ulipristal by an external physician for an erroneously presumed diagnosis of fibroids were found to have adenomyosis after reporting an increase in pelvic pain post-treatment [42]. Enhancement of adenomyotic features on ultrasound after treatment was also noted when compared to the external scans done at diagnosis [42]. A similar recent study was also published by Calderon et al. showing progression of adenomyosis after treatment with ulipristal for concurrent fibroids in 12 out of 15 patients (80%) who had adenomyotic features on pre-treatment MRIs and development of novel adenomyosis in 15 out of 57 patients (26.3%) with no pre-treatment evidence of adenomyosis on MRI [43]. This data suggests that ulipristal acetate could enhance progression or provoke emergence of adenomyosis. Adverse effects of ulipristal acetate treatment are amenorrhea, weight gain, fatigue, abdominal discomfort, decreased menstrual flow, dizziness, facial flushing, dry eyes, headache, breast discomfort, and increased vaginal discharge [44]. Liver injury requiring the need for liver transplantation has been reported as an adverse effect of this drug as well, which led to its withdrawal from the European market, limiting the accessibility of this drug in the region [45].

#### 3.1.9. Antiplatelet Therapy

A study on mice by Zhu et al. suggested that antiplatelet therapy suppressed myometrial infiltration, improved generalized hyperalgesia and reduced uterine contractility in mice in which adenomyosis has been induced [46]. More studies are needed to evaluate the role of antiplatelets on adenomyosis therapy and as far as we know there are no human data.

#### 3.1.10. Dopamine Agonists

The effect of bromocriptine, a dopamine agonist and prolactin inhibitor, has been evaluated in women diagnosed with adenomyosis in 2 studies by Andersson et al. [42,43]. The effect of prolactin is unclear in the pathogenesis of adenomyosis but prolactin and its receptors seem to increase in adenomyotic tissue which suggests an association between the hormone and the disease [47]. A pilot study by Andersson et al. evaluated treatment with vaginal bromocriptine for 9 months in 19 patients with adenomyosis. Women who received vaginal bromocriptine therapy reported significantly lower 9 months scores on PBAC (baseline 349 vs. 9-month mark 233, *p* = 0.003), visual analog scale (5 vs. 2.5, *p* < 0.001), endometriosis health profile (EHP) core pain (15.9 vs. 3.4, *p* = 0.029), EHP core self-image (41.7 vs. 25, *p* = 0.048), symptom severity score (60 vs. 44, *p* < 0.001), and higher health-related quality of life scores (57 vs. 72, *p* < 0.001) [48]. In another study Andersson et al. also evaluated bromocriptine using imaging. They showed a thinner maximal junctional zone (8.5 vs. 7.9 mm, *p* = 0.02) at 6 months on ultrasound but results were not significant on MRI (16 vs. 15.5 mm, *p* = 0.81) [49]. Asymmetric wall thickening was seen in 72% of patients on at baseline and only in 33% at 6 months post treatment. No changes were noted in irregular endometrial-myometrial border, presence of fan-shaped shadowing, cystic changes, striations, hyperechogenic islands or lesion extension [49].

#### 3.1.11. Oxytocin Antagonists

Oxytocin antagonists are being investigated for the use in adenomyosis treatment because overexpression of oxytocin receptor has been demonstrated in uteri with adenomyosis [50]. In a phase I trial, Epelsiban, a selective oxytocin receptor antagonist, was tested on a population of healthy women and was found to be well tolerated with no significant safety concerns [51]. Further trials on patients with adenomyosis are needed to evaluate the efficacy.

### 3.2. Surgical & Procedural Management (for Diffuse or Adenomyoma Specify)

#### 3.2.1. Hysterectomy

Historically, hysterectomy was considered the definitive diagnostic and therapeutic approach to adenomyosis given that it removes the source of pathology. However, with the increasing numbers of younger patients with adenomyosis, treatments that preserve fertility emerged to avoid surgical removal of the uterus [5]. It is currently an acceptable treatment option for when other, more conservative therapies have failed. The cervix can be retained if no cervical patholy necessitates its removal as a supracervical hysterectomy has not been shown to increase the risk of symptom persistence when compared to a total hysterectomy in patients with adenomyosis [52].

#### 3.2.2. Uterus-Sparing Resection

Uterus-sparing surgical approaches were developed for the treatment of adenomyosis. These methods are primarily based on the principle of removing the diseased tissue to decrease uterine size and improve clinical symptoms. Wood et al. used conservative surgical techniques to treat 14 patients with adenomyosis, and reported marked improvement of menorrhagia and dysmenorrhea in 4 of 7 patients after endometrial resection, 3 of 4 after myometrial reduction, and all 3 who underwent myometrial excision [53]. Saremi et al. investigated a novel technique of adenomyomectomy in 103 women with adenomyosis who wished to preserve fertility. Post-surgical treatment, 65% of the patients experienced a decrease in abnormal uterine bleeding and 41% reported a decrease in dysmenorrhea [54]. In another study by Shu et al., outcomes of wedge-shaped resection of uterine adenomyosis were analyzed in 15 patients. This procedure was shown to be safe and effective in reducing menorrhagia and alleviating the extent dysmenorrhea [55]. A systematic review by Grimbizis et al. found that uterine-sparing operative treatment are feasible and efficacious in treatment of adenomyosis, such that complete excision reduced the dysmenorrhea rate by 82%, controlled menorrhagia by 68.8%, and increased pregnancy rate to 60.5% [56]. After partial excision, dysmenorrhea reduction, menorrhagia control, and pregnancy rates were 81.8%, 50% and 46.9%, respectively [56]. In another systematic review by Younes et al., 25 to 80% of patients had reduction in menorrhagia and 50 to 94.7% had pain improvement after complete excision [57]. After incomplete excision, 40% reported improvement in menorrhagia and 55–94% had pain improvement [57]. In non-excisional techniques such as endometrial ablation and myometrial electrocoagulation, a comparable proportion of 57 to 86.6% of patients had pain control and 81.3 to 98.4% had bleeding control. Recurrence rate was estimated to be 9% in complete excision, 19% in partial excision, and 32.5% in non-excisional techniques [57]. Partial resection combined with uterine artery occlusion was also studied by Kang et al. on a total of 37 patients with adenomyosis, and was also noted to be effective in reducing menorrhagia (post-op menorrhagia score was 59 at 12 months compared to 158 at baseline, *p* < 0.001), dysmenorrhea (12 months post-op score of 4 vs. 8 pre-op, *p* < 0.001) and uterine volume (91.6 cm^3^ at 12 months post-op vs. 224.6 cm^3^, shrinkage rate of 59.2% *p* < 0.001) [58]. Furthermore, Zheng et al. found that resection combined with LNG-IUD was more effective in reducing menstrual flow when compared to the IUD alone (*p* < 0.001), but no difference in pain reduction was noted (*p* = 0.061) [59]. A case report by Ota et al. was recently published investigating a new technique using real-time intraoperative ultrasound elastography guidance during laparoscopic resection of adenomyosis to preserve healthy uterine tissue while avoiding residual disease in the myometrium [60].

#### 3.2.3. Uterine Artery Embolization (UAE)

Uterine artery embolization has been used in the treatment of leiomyomas and was shown to be as effective as myomectomy in improving quality of life and controlling symptoms [61]. Its use in both diffuse and focal adenomyosis is being investigated and studies are promising so far. Siskin et al. performed a retrospective review of 15 patients with adenomyosis and menorrhagia who underwent UAE and were followed up with imaging. At 12-months follow-up, 92.3% of patients reported significant improvement in presenting symptoms and quality of life [62]. MR imaging performed at a mean of 6 months post treatment revealed significant reductions in median uterine volume (42%) and mean junctional zone thickness (11 mm, 33%, *p* < 0.5) [62]. Significant improvement in dysmenorrhea (95.2%) and menorrhagia (95%) were also reported by Kim et al., along with coagulation necrosis of adenomyosis (72.1% of patients), decreased size without necrosis (25.6% of patients), and a mean uterine volume reduction of 32.5% on MRI [63].

Other studies reported similar results consistent with a decrease in uterine volume on MRI and a significant reduction in menorrhagia and dysmenorrhea [64,65,66,67,68]. Smeets et al. assessed long-term outcomes of UAE at 65 months post treatment and found that UAE resulted in long-term preservation of the uterus without clinical symptoms. They reported that the only predictor for hysterectomy was the initial thickness of the junction zone [69]. In another 7 years clinical follow up by de Bruijn et al., 82% of UAE-treated patients with adenomyosis avoided a hysterectomy and 72% of them reported being fairly satisfied about the treatment [70]. Quality of life and symptom severity scores were significantly decreased 3 months post treatment and were still comparable at the 7 years follow up [70]. The current ongoing QUESTA trial is comparing UAE to hysterectomy in patients with adenomyosis and should provide further evidence for the use of UAE as a treatment option [71]. The impact of UAE on infertility is still not well established and should also be taken into consideration [72].

#### 3.2.4. Radiofrequency Ablation

Radiofrequency ablation of adenomyotic lesions is another promising uterine-preserving option for focal adenomyosis. Scarperi et al. found a significant reduction in adenomyosis volume (24.2 vs. 60 cm^3^, *p* < 0.01) and a 68.1% visual analog scale score reduction at 9 months post laparoscopic radiofrequency ablation [73]. Hai et al. performed ultrasound-guided transcervical radiofrequency ablation for adenomyosis on 87 patients and reported a 41.2% uterine volume reduction and a focal adenomyosis volume decrease of 54.7% at 12 months post treatment [74]. The VAS scores significantly declined from 6.9 to 1.9 at 12 months follow up and symptoms severity score also showed a drop from 44 to 11.85 at 12 months (*p* < 0.001) [74]. Radiofrequency ablation was also shown to maintain fertility with a pregnancy success rate reaching up to 50% making it a desirable option for patients who wish to conceive [75]. Radiofrequency ablation is also effective when combined with LNG-IUD demonstrated by a reduction of uterine volume and a decrease in dysmenorrhea [76].

#### 3.2.5. High-Intensity Focused Ultrasound (HIFU)

High-intensity focused ultrasound is a non-surgical option that utilizes ultrasound waves to cause coagulative necrosis and cell death to pathologic tissue [77]. It has been widely studied in the treatment of adenomyosis and results were consistent in revealing that it is safe and effective but RCTs are lacking. HIFU showed a significant decrease in dysmenorrhea scores and in volume of adenomyotic lesions across several studies [78,79,80,81]. Menorrhagia also seems to significantly improve after treatment with HIFU in both focal and diffuse adenomyosis [82,83,84]. Results were sustained at 2 years follow up as shown by Shui et al. such that the dysmenorrhea relief rate was 82.3% and menorrhagia relief rate reached 78.9% at 2 years post treatment [85]. In a meta-analysis by Marques et al., pooled results showed a significant reduction in uterine volume (standard mean difference, SMD = 0.85), dysmenorrhea (SMD = 2.37), and significant improvement in quality of life (SMD = 2.75) at 12 months post treatment of adenomyosis with HIFU [86]. The effect on HIFU on fertility has not been well established. In a study by Lee et al., levels of anti-Mullerian hormone were measured pre and post treatment to assess the impact of this treatment on ovarian reserve and found no significant difference between levels pre-treatment and at 6 months post-treatment (2.11 vs. 1.84, *p* > 0.05) suggesting that HIFU has no effect on ovarian function [87]. When compared to laparoscopic excision, HIFU showed significantly higher pregnancy and natural conception rates and was comparable to excision in terms of pain and menorrhagia reduction [88]. Further studies are required to determine fertility outcomes. HIFU was also studied in combination with GnRH agonists or with LNG-IUD and combination therapy was noted to be more effective than treatment with HIFU alone, especially when comparing long term outcomes [89,90]. Uterine and adenomyotic volume, dysmenorrhea, and menorrhagia all significantly decreased upon addition of GnRH after HIFU treatment (*p* < 0.05) [91]. Similar results were seen when HIFU was combined with LNG-IUD and long-term efficacy also seemed to be enhanced in the combined treatment such that the 6 months and 3 years follow up results were significantly higher in groups treated with combination therapy than with HIFU alone [92,93].

## 4. Conclusions

Treatment of adenomyosis varies widely from simple medication to a total hysterectomy and several options in between. Randomized controlled trials that compare treatments are insufficient but there is a growing body of evidence to support the use of several emerging therapeutic approaches such as GnRH agonist and antagonist therapy, progestin IUDs and ultrasound ablation. Further studies are needed to determine fertility outcomes and long-term effects of these treatments. To date, the choice of therapy for adenomyosis is still individualized and should rely on the clinical presentation of patients and the desire for future pregnancy, especially with the increasing number of nulliparous, younger patients with this condition. Levonorgestrel IUD is effective, non-invasive, and fertility-preserving and seems to be the superior choice of treatment for this population. However, further evidence is still required to establish definite treatment guidelines for adenomyosis.

## Data Availability

Not applicable.
